# Phosphodiesterase 4 is overexpressed in keloid epidermal scars and its inhibition reduces keratinocyte fibrotic alterations

**DOI:** 10.1186/s10020-024-00906-8

**Published:** 2024-09-02

**Authors:** Javier Milara, Pilar Ribera, Severiano Marín, Paula Montero, Inés Roger, Julio Cortijo

**Affiliations:** 1 CIBER de enfermedades respiratorias, Health Institute Carlos III, Valencia, Spain; 2 Department of Pharmacology, Faculty of Medicine, University of Valencia, Avenida Blasco Ibáñez, 15, Valencia, 46010 Spain; 3 Pharmacy unit, University General Hospital Consortium of Valencia, Valencia, Spain; 4 Plastic Surgery Unit, University General Hospital Consortium, Valencia, 46014 Spain; 5 Faculty of health sciences, Universidad Europea de Valencia, 46010 Valencia, Spain

**Keywords:** Skin fibrosis, Keloid, Hypertrophic scar, Phosphodiesterase 4

## Abstract

**Background:**

Epidermal remodeling and hypertrophy are hallmarks of skin fibrotic disorders, and keratinocyte to mesenchymal (EMT)-like transformations drive epidermis alteration in skin fibrosis such as keloids and hypertrophic scars (HTS). While phosphodiesterase 4 (PDE4) inhibitors have shown effectiveness in various fibrotic disorders, their role in skin fibrosis is not fully understood. This study aimed to explore the specific role of PDE4B in epidermal remodeling and hypertrophy seen in skin fibrosis.

**Methods:**

In vitro experiments examined the effects of inhibiting PDE4A-D (with Roflumilast) or PDE4B (with siRNA) on TGFβ1-induced EMT differentiation and dedifferentiation in human 3D epidermis. In vivo studies investigated the impact of PDE4 inhibition on HOCl-induced skin fibrosis and epidermal hypertrophy in mice, employing both preventive and therapeutic approaches.

**Results:**

The study found increased levels of PDE4B (mRNA, protein) in keloids > HTS compared to healthy epidermis, as well as in TGFβ-stimulated 3D epidermis. Keloids and HTS epidermis exhibited elevated levels of collagen Iα1, fibronectin, αSMA, N-cadherin, and NOX4 mRNA, along with decreased levels of E-cadherin and ZO-1, confirming an EMT process. Inhibition of both PDE4A-D and PDE4B prevented TGFβ1-induced Smad3 and ERK1/2 phosphorylation and mesenchymal differentiation in vitro. PDE4A-D inhibition also promoted mesenchymal dedifferentiation and reduced TGFβ1-induced ROS and keratinocyte senescence by rescuing PPM1A, a Smad3 phosphatase. In vivo, PDE4 inhibition mitigated HOCl-induced epidermal hypertrophy in mice in both preventive and therapeutic settings.

**Conclusions:**

Overall, the study supports the potential of PDE4 inhibitors, particularly PDE4B, in treating skin fibrosis, including keloids and HTS, shedding light on their functional role in this condition.

**Supplementary Information:**

The online version contains supplementary material available at 10.1186/s10020-024-00906-8.

## Background

Skin fibrosis is characterized by an aberrant scarring followed by unregulated extracellular matrix (ECM) deposition in the dermis, leading to a compromised function and altered structure (1). Skin fibrosis is related to a wide array of dermatological diseases, such as pathological scars, including hypertrophic scars and keloids, or systemic sclerosis (SSc) among others (Andrews et al. 2016; Rockey et al. 2015), affecting to the patients’ quality of life, with cosmetic harm, pruritus, pain, organ dysfunction, psychological distress or even with life-threatening consequences (Mura et al. 2012; Wynn 2008).

Cellular injury and chronic inflammation are considered key underlying causes of fibrotic tissue remodeling, which is characterized by subsequent activation of inflammatory cells, oxidative stress and uncontrolled effector cells activation (Rockey et al. 2015). Activated fibroblasts and myofibroblasts are the central effector cells of the fibrotic process, being the main producers of ECM. Myofibroblasts, the main producers of collagen, can originate from resident fibroblasts, epithelial and endothelial cells (epithelial/endothelial-mesenchymal transition; EMT/EndMT), bone marrow derived fibrocytes, pericytes or adipocytes (Do and Eming 2016; Wynn 2008).

Recent studies indicates that keloid keratinocytes can be under EMT process, showing decreased expression of epithelial markers an increased number of mesenchymal markers accompanied by elevated migratory capacity and myofibroblast characteristics which support an important role on skin fibrosis (Yuan et al. 2019).

Several pro-fibrotic growth factors have been described to play a role in skin fibrosis (Rockey et al. 2015). Particularly, transforming growth factor beta (TFGβ) is considered a key regulator of fibrosis and is known to activate multiple differentiation pathways, ECM production, and oxidative stress-related genes involved in profibrotic pathways among others (Samarakoon et al. 2013; Shroff et al. 2014). However direct target therapies against TFGβ failed due to different toxicities (Aluwihare et al. 2009; Morris et al. 2003; Sheppard 2001; Yang et al. 2007), so new approaches are needed to understand skin fibrosis pathogenesis and treatments.

Adenosine 3´,5´-cyclic monophosphate (cAMP) is a second messenger molecule that modulates a broad number of cellular processes in response to pro-inflammatory and pro-fibrotic agents (Cortijo et al. 2009; Milara et al. 2014a, b). The levels of cAMP are controlled by phosphodiesterase 4 (PDE4), a family of enzymes that degrade cAMP to monomeric AMP, thereby inactivating the molecule.

The PDE4 family is composed of four subtypes (PDE4A-D) encoded by different genes that by alternative splicing are expressed as multiple variants differing in their N-terminal domains (Conti et al. 2003; Houslay et al. 2005). PDE4 plays a role in almost all inflammatory cells, reducing monocyte-macrophage activation and secretion of cytokines and chemokines, inhibiting neutrophil chemotaxis and degranulation and lymphocyte chemotaxis and proliferation. In addition, in fibroblasts inhibits fibroblast proliferation and myofibroblast transformation, and in epithelial cells reduces epithelial secretion of pro-inflammatory mediators (Hatzelmann et al. 2010). In this regard, PDE4 inhibition reduces fibrotic markers, activation of lung fibroblasts and EMT (Milara et al. 2014a, b; Vecchio et al. 2013) as well as lung fibrosis in an animal model of intratracheal bleomycin (Cortijo et al. 2009). In this line, recent studies indicate that the PDE4 inhibitor apremilast, reduces the skin fibroblast to myofibroblast transition induced by TGFβ in vitro, as a part of the skin fibrotic process (Cutolo et al. 2020; Schafer et al. 2016). Furthermore, apremilast ameliorates the pathological manifestations of SSc in different animal models, including skin dermal thickness, deposition of collagens, increased expression of α-SMA and activation of M1/2 macrophages and T cells (Lu et al. 2021; Maier et al. 2017). However, to our knowledge there is no data regarding the role of PDE4 in fibrotic skin lesions such as hypertrophic scars and keloids, and no data exist about the role of PDE4 inhibition on keratinocyte activation and transformation into mesenchymal cells as part of the pathological scar development. Here we analysed for the first time the expression of PDE4 isoforms on the epidermis of hypertrophic scars and keloids, and the role of PDE4 on the keratinocyte activation in vitro and in vivo in order to provide new evidence on the potential role of PDE4 inhibitors on the skin fibrotic process.

## Methods

### Human skin tissue

A total of 8 patients (6 women and 2 men) with keloids, and 8 patients (5 women and 3 men) with hypertrophic scar, were recruited to the present study. Keloid and hypertrophic scar specimens were obtained from these patients, which were aged between 35 and 56 years old for keloid and, ranging 31 to 51 years old for hypertrophic scar. Patients did not show systemic diseases.

Inclusion criteria included skin lesions diagnosed as keloids or hypertrophic scar according to clinical appearance, symptoms, persistence for > 1 year and extension beyond the original margins (keloids) or within the borders of the original wound (hypertrophic scars). All of the patients’ keloids and hypertrophic scars were in the active stage and none had undergone prior treatment. Prior to surgery, all patients were informed of the purpose and procedure of the present study and agreed to provide their resected lesion masses. The whole keloid/ hypertrophic scar tissues were completely removed, following administration of local anesthesia, from the skin of the neck, chest and abdomen, according to the standard surgical procedures of the Plastic Surgery Unit of the *Consorcio Hospital General Universitario de Valencia* CHGUV, Spain. Human healthy skin tissue (control samples) was obtained from patients undergoing aesthetic surgery procedures / without any skin disease (*n* = 8). Data from patients included in this work is showed in additional table S1. The protocol was approved by the local research and independent ethics committee of the CHGUV (CHGUV/16/1/2016) and performed according to the Declaration of Helsinki. Informed written consent was obtained from each participant.

### Cell cultures

Primary keratinocytes were obtained from healthy, hypertrophic scar and keloid epidermis. For each donor, one piece of skin was digested with dispase (5 U/ml, STEMCELL Technologies, Vancouver, Canada) overnight at 4 °C to detach the epidermis from the dermis, as previously described (Talabot-Ayer et al. 2019). Epidermis layer was placed onto a cell culture plate in Keratinocyte Growth Medium (KGM-Gold™, Lonza Basel, Switzerland) supplemented with KGM-Gold SingleQuot Kit (Lonza) until keratinocytes were observed. Cell experiments were done until passage 3.

To create a 3D epidermis model, primary adult epidermal keratinocytes (192627, Lonza, Basel, Switzerland) were cultured at 37ºC and 95% air/5% CO_2_ using Keratinocyte Growth Medium (KGM-Gold™, Lonza Basel, Switzerland) supplemented with KGM-Gold SingleQuot Kit (Lonza) in monolayers. 3D epidermis cell models were reconstructed using the BALB/3T3 feeder-layer technique adapted from Mak et al. (Mak et al. 1991) and Arnette et al. (Arnette et al. 2016). In brief, 10^6^ BALB / 3T3 fibroblasts (Lonza, Basel, Switzerland) were seeded on collagen-coated Millicell inserts (Millicell-CM 12 mm, transparent Biophore Membrane; Millipore CORP., Bedford) and placed into 6-well plates (Corning Incorporated; Corning, U.S.). Fibroblasts were cultured for 2 days in 1 ml Dulbecco’s Modified Eagle Medium (DMEM, high glucose; Gibco, U.S.) supplemented with 10% fetal calf serum (FCS, Gibco, U.S.) and added to the apical and dorsal side of the insert. When fibroblasts reached 60–70% confluence, the monolayer was irradiated with UV light at 0.048 mW for 1 h with UVACUBE 400 (Honle UV Technology, Germany) to establish the feeder layer. Then, primary adult epidermal keratinocytes (192627, Lonza, Basel, Switzerland) were seeded at a density of 0.5 × 10^6^ cells/cm^2^. Cultures were grown at 37ºC and 95% air/5% CO_2_ until approximately 60% confluency and then were switched to Keratinocyte Growth Medium (KGM-Gold™, Lonza Basel, Switzerland) supplemented with KGM-Gold SingleQuot Kit (Lonza) until confluent. Confluent cultures were raised to the air-liquid interface and cultured for 21 days until epidermal stratification was achieved. To validate the stratification, histological analysis was performed after 21 days. The reconstructed epidermis tissues were fixed with 10% formalin solution, dehydrated, and embedded in paraffin. Six-micrometer-thick sections were cut and stained with hematoxylin-eosin. Random photographs were taken of each sample with a Leica DM6000B microscope (Leica Biosystems; Wetzlar, Germany).

### Histological, Immunohistochemical and immunofluorescence studies

To measure mouse skin thickness in the HOCL animal model, histology was done as previously reported (Peiro et al. 2022). Briefly, skin tissue blocks (4 μm thickness) were stained with hematoxylin & eosin. Ten random measurements were taken per section. Two investigators examined all the sections independently in a blinded fashion. The results were expressed in millimeters and summarized as mean (± SEM) values of epidermal thickness for each group.

Immunofluorescence analysis was performed to detect PDE4B2, p-ERK1/2, αSMA and NOX4 expression and distribution in human and mice skin tissue. Skin tissue was fixed in paraformaldehyde (4%) for 48 h, and tissue was embedded in Tissue-Tek^®^ OCT™ cryosectioning compound (Sakura Finetek Europe BV, Leiden). Blocks were cut into 10 μm thick sections, permeabilized in Triton X 100 (0.1% in PBS) for 5 min, blocked in 10% goat serum in PBS and immunostained with monoclonal mouse or polyclonal rabbit antibodies against human or mouse PDE4B2 (Sigma-Aldrich, cat nº ABS181; RRID: AB_10851821), phospho-ERK1/2 (Sigma Aldrich, cat. nº M9692; RRID: AB_260729), total ERK1/2 (Cell Signaling, cat. n. 4695; RRID: AB_390779), αSMA (Sigma-Aldrich, cat. nº A5228; RRID: AB_262054) and NOX4 (Novus, cat. nº NB110-58849; RRID: AB_877739) for 24 h at 4ºC followed by a secondary FITC or rhodamine conjugated anti-mouse/rabbit IgG antibody and finally DAPI (2 µg/ml) to mark nuclei (Molecular Probes, Leiden, The Netherlands). Co-localization of PDE4B2/αSMA and PDE4B2/p-ERK1/2 were performed using a confocal spectral Leica TCS SP2 microscope with ×600 magnification and 3× zoom. Red (HeNe 543 nm), green (HeNe 488 nm), and blue (Ar 351 nm, 364 nm) lasers were used. Co-localization studies were performed using the Leica confocal software v2.61. The cell images with co-localized points of the two laser canals were transformed into an orange color in the image. The number of epidermal α-SMA-positive cells in each section, were counted in three randomly chosen high-power fields (HPF) at a magnification of 200-fold in a blinded fashion.

Immunofluorescence quantification was conducted with MetaMorph^®^ Software (Molecular Devices, USA). The epidermal layer was selected in 10 different folds per patient at 20x magnification, maintaining consistent excitation and exposure times across all conditions, in a blinded manner. Fluorescence intensity was measured in relative fluorescence units (RFU).

### In vitro experimental conditions

For in vitro studies, primary keratinocytes monolayers or 3D keratinocytes were stimulated with TGFβ1 at 10ng/ml for the indicated times, replacing culture medium and stimulus every 24 h. Roflumilast (PDE4 inhibitor, 100 nM; Takeda, Osaka, Japan), PD98059 (ERK inhibitor, 10µM, Sigma-Aldrich), N-acetyl-L-cysteine (NAC, anti-oxidant, 1mM, Sigma-Aldrich), SIS3 (SMAD3 inhibitor, 10µM, Sigma-Aldrich), KT5720 (PKA inhibitor, 2µM, Sigma-Aldrich), sanguinarine (PPMA1 inhibitor, 3 µM, Sigma-Aldrich) or vehicle (0.1% DMSO) was added 30 min before stimulus. In 3D keratinocyte cultures, both test compounds and TGFβ1 were added to the basolateral media (500 µl) and at the apical surface (25 µl). Drug concentrations were established based on their inhibitory concentrations 50 (IC50) (Davies et al. 2000; Hatzelmann and Schudt 2001; Iwasaki et al. 2016; Kim et al. 2011; Zhang and Huang 2019).

### Western blotting analysis

Western blotting analysis was used to detect changes in human skin keratinocytes and mice skin tissues protein expression. Tissue/cells were homogenized or scraped from a confluent 25-cm^2^ flask and lysed on ice with a lysis buffer comprising a complete inhibitor cocktail plus 1 mM ethylenediaminetetraacectic acid (Roche Diagnostics Ltd., West Sussex, UK) with 20 mM Tris base, 0.9% NaCl, 0.1% Triton X-100, 1 mM dithiothreitol, and 1 mg/mL pepstatin A. The Bio-Rad assay (Bio-Rad Laboratories Ltd., Herts, UK) was used according to the manufacturer’s instructions to quantify the level of protein in each sample to ensure equal protein loading. Sodium dodecyl sulfate polyacrylamide gel electrophoresis was used to separate the proteins according to their molecular weight. Briefly, 15 µg of proteins (denatured) along with a molecular weight protein marker (Bio-Rad Kaleidoscope marker; Bio-Rad Laboratories) were loaded onto an acrylamide gel consisting of a 5% acrylamide stacking gel stacked on top of a 10% acrylamide resolving gel and run through the gel by application of 100 V for 1 h. Proteins were transferred from the gel to a polyvinylidene difluoride membrane using a wet-blotting method. The membrane was blocked with 5% Marvel in PBS containing 0.1% Tween20 (PBS-T), probed with the following antibodies: PDE4B2 (Sigma-Aldrich, cat nº ABS181), phospho-SMAD3 (Novus Biologicals, cat.nº NBP1-77836; RRID: AB_11031542), phospho-ERK1/2 (Sigma Aldrich, cat.nº M9692), αSMA (Sigma-Aldrich, cat. nº A5228), collagen type I (Novus Bilogicals, cat.nº NB600-408; RRID: AB_10000511), PPM1A (Cell signaling, cat. nº 3549 S; RRID: AB_2169764), phospho-PDE4B/D ERK site antibody (FabGennix, cat, nº PPD4-450AP; RRID: AB_3095755), E-cadherin (ECM Bioscience, cat, nº CM1681; RRID: AB_2076812), NOX4 (Novus, cat. nº NB110-58849; RRID: AB_877739) and normalized to total anti-human/mouse β-actin (1:1000) antibody (42 KDa, monoclonal antibody, catalog no. A1978; RRID: AB_476692, Sigma Aldrich). The enhanced chemiluminescence method of protein detection using enhanced chemiluminescence reagents (ECL Plus; Amersham GE Healthcare, Buckinghamshire, UK) was used to detect labeled proteins. Densitometry of films was performed using the Image J 1.42q software (available at http://rsb.info.nih.gov/ij/, USA). Results of target protein expression are expressed as the percentage of the densitometry of the endogenous controls β-actin.

### Immunoprecipitation

Equal amounts of protein (300 µg) from total protein extracts were incubated with 2 µg of p-ERK1/2 antibody and the IgG isotype control. The immune complexes were precipitated with protein G on Sepharose 4B fast flow beads (Sigma Aldrich; catalogue no. P-3296) overnight at 4 °C. After washing three times with NET buffer containing 50 mM Tris-HCl at pH 8.0, 150 mM NaCl, and 0.1% Nonidet P-40, the bound materials were eluted from the immunoprecipitates in reducing SDS-PAGE loading buffer containing 10% SDS, 1 M Tris-HCl at pH 6.8, 50% glycerol, 10% 2-mercaptoethanol, and 2% bromophenol blue at 100 °C for 10 min. Immunoprecipitated protein complexes were assayed by western blotting as described above and probed using PDE4B2 or p-ERK1/2 antibodies, as appropriate.

### Real-time RT-PCR and siRNA experiments

Total RNA was isolated using TriPure^®^ Isolation Reagent (Roche, Indianapolis, USA). The integrity of the extracted RNA was confirmed with Bioanalyzer (Agilent, Palo Alto, CA, USA). Reverse transcription was performed in 300 ng of total RNA with a TaqMan reverse transcription reagents kit (Applied Biosystems, Perkin-Elmer Corporation, CA, USA, cat.nº N8080234). cDNA was amplified with specific primers and probes predesigned by Applied Biosystems for human PDE4A (Hs00183479_m1), PDE4B (Hs00387320_m1), PDE4C (Hs00971865_m1), PDE4D (Hs00174805_m1), α-SMA (Hs00559403_m1), α1(I)-collagen (collagen type I; Hs00164004_m1), p21 (Hs01040810_m1), fibronectin (Hs01549976_m1), E-cadherin (Hs01023894_m1), zona occludens-1 (ZO-1) (Hs01551861_m1) and N-cadherin (Hs00983056_m1) in a 7900HT Fast Real-Time PCR System (Applied Biosystems) using Universal Master Mix (Applied Biosystems). Expression of the target gene was expressed as the fold increase or decrease relative to the expression of β-actin as an endogenous control (Applied Biosystems; Hs01060665). The mean value of the replicates for each sample was calculated and expressed as the cycle threshold (Ct). The level of gene expression was then calculated as the difference (ΔCt) between the Ct value of the target gene and the Ct value of β-actin. The fold changes in the target gene mRNA levels were designated 2^-ΔCt^.

Small interfering RNA (siRNA), including the scrambled siRNA control, was purchased from Thermo Fisher Scientific (Huntingdon, Cambridge, UK; catalogue no. 4390843). PDE4B gene-targeted siRNA (identification no. 11667), was designed by Thermo Fisher Scientific. Cells were transfected with siRNA (50 nM) in serum and antibiotic-free medium. After 6 h, the medium was aspirated and replaced with medium containing serum for a further 42 h before cell stimulation. The transfection reagent used was lipofectamine-2000 (Invitrogen, Paisley, UK; catalogue no. 11668-027) at a final concentration of 2 µg/mL.

### Proliferation

Keratinocyte proliferation was measured by colorimetric immunoassay based on BrdU incorporation during DNA synthesis using a cell proliferation enzyme-linked immunosorbent assay BrdU kit (Sigma Aldrich, cat. nº 11647229001) according to the manufacturer’s protocol. Cells were seeded at a density of 3 × 10^3^ cells/well on 96-well plates and incubated for 24 h. TGFβ1, PDGF or FGF stimulation was incubated at indicated times. The 490 nm absorbance was quantified using a microplate spectrophotometer (Victor 1420 Multilabel Counter, PerkinElmer). Proliferation data refer to the absorbance values of BrdU-labeled cellular DNA content per well.

### DCF Fluorescence Measurement of reactive oxygen species

2´, 7´-dichlorodihydrofluorescein diacetate (H_2_DCF-DA, Molecular Probes, UK) is a cell-permeable compound that following intracellular ester hydrolysis is oxidized to fluorescent 2´, 7´-dichlorofluorescein (DCF) by O_2_^**·**−^ and H_2_O_2_, and can therefore be used to monitor intracellular generation of reactive oxygen species (ROS) (Trayner et al. 1995). To quantify ROS levels, keratinocytes were washed twice with PBS and incubated for 30 min with 50 µM H_2_DCF-DA diluted in Opti-MEM in presence of roflumilast or vehicle. Then, cells were again washed twice with PBS to remove remaining H_2_DCF-DA and stimulated with TGFβ for 30 min in presence of roflumilast or vehicle. Five randomly selected fields per condition were measured for fluorescent intensity using an epifluorescence microscope (Nikon Eclipse TE 200, Tokio, Japan) with filter set for FITC. Subsequent image capture and analysis was performed using Metafluor^®^ 5.0 software (Analytical Technologies, US). Results were expressed as DCF fluorescence in relative fluorescence units.

### Fibrosis animal model

Experimentation and handling were performance in accordance with the guidelines of the Committee of Animal Ethics and Well-being of the University of Valencia (Valencia, Spain; 2017/VSC/PEA/00062). The animal studies followed the ARRIVE guidelines (Kilkenny et al. 2010). The in vivo mouse model was previously established in our laboratory (Peiro et al. 2022). Pathogen-free male Balb/c (Charles River Laboratory) mice of 12 weeks of age, body weight between 15 and 22 g, were used in the experiments.

In the Cochin chronic oxidant stress model (Servettaz et al. 2009) skin fibrosis developed secondary to the subcutaneous, once daily administration of hypochlorous acid (HOCl) to Balb/c mice (Bagnato et al. 2013). Following previously described protocols (Bitto et al. 2016), hypochlorous acid (HOCl) at a concentration of 2.5 µM was generated by adding 166 µl of sodium hypochlorite (NaClO) solution (2.6% as active chlorine) to 11.1 ml of potassium dihydrogen phosphate KH_2_PO_4_ solution (100 mM, pH 7.2).

Twenty-four mice were randomly allocated to three treatment groups (i) control (sham), (ii) vehicle / HOCl and (iii) roflumilast / HOCl at *N* = 8 animals per group.

Mice allocated to treatment groups (ii) and (iii) were subjected to once daily, subcutaneous administrations of HOCl at 2.5 µM concentration in an injection volume of 100 µl corresponding to 0.25 nmol HOCl per mouse and day for 6 weeks. Blinded mice allocated to treatment group (i) (Sham) received once daily, subcutaneous administration of saline (0.9% NaCl) in a 100 µl injection volume. Subcutaneous injections were done into the back of the mouse using a 27 G needle.

To minimize the procedural burden to the animals, the administrations were carried out under anesthesia with inhaled isoflurane (Aerrane^®^) 5%.

Roflumilast was administered once daily, orally by gavage at 5 mg / kg / d (Treatment group iii) from a suspension formulation composed of methocel and PEG400 and at a dose volume of 10 ml / kg based in previous antifibrotic experiments (Cortijo et al. 2009). An identical formulation but without roflumilast was administered in treatment group (ii) (Vehicle).

For preventive treatment, roflumilast was administered from day 1 to day 42. For therapeutic treatment, the PDE4 inhibitor was administered from day 20 to 42.

The animals were sacrificed by intraperitoneal administration of pentobarbital 35 mg / kg. Skin samples were taken and fixed in formaldehyde for histology purposes.

### Statistical analysis

Statistical analysis of results was carried out by non-parametric analysis. *P* < 0.05 was considered statistically significant. Data were displayed as medians, interquartile range, and minimum and maximum values. When the comparisons were either between only 2 groups or between more groups, between-group differences were analyzed by either the Mann Whitney or Kruskal-Wallis test followed by the Dunn’s post-hoc test.

## Results

**Fig. 1:**
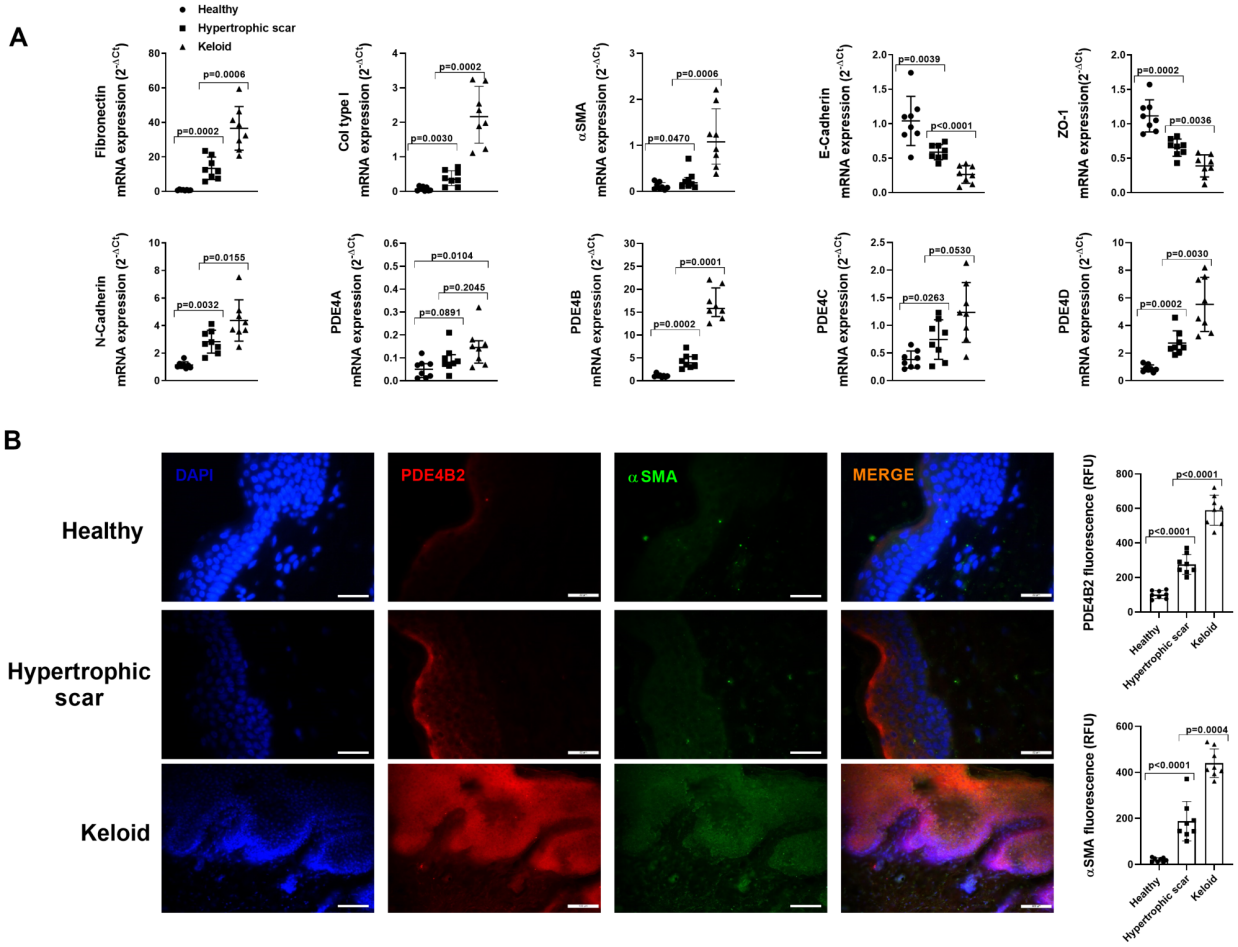
Epidermal expression of PDE4 isoforms in healthy skin, hypertrophic scar and keloids. Epidermal biopsies from dermatologically healthy skin (*N* = 8), hypertrophic scars (*N* = 8) and keloid (*N* = 8) were processed to extract RNA, or fixed for immunofluorescence. (**A**) mRNA expression of PDE4 **A**, **B**, **C** and **D** isoforms, the fibrotic markers collagen type I, fibronectin, αSMA and N-cadherin, and the epithelial markers E-cadherin and ZO-1 measured by RT-qPCR and expressed as 2^−ΔCt^ of the diseased epidermis versus healthy epidermis. (**C**) Immunofluorescence of PDE4B2 (red) and αSMA (green), their co-expression (orange) and fluorescence intensity measured as relative fluoresce units (RFU) in the epidermal layer. Representative images are shown. Scale bar: 100 μm. Data are presented as scatter dot blot with median and interquartile range values. P-values are based on Kruskal-Wallis test followed by the Dunn’s post-hoc test

Keratinocyte epidermal cells were isolated from healthy (*n* = 8), hypertrophic scar (*n* = 8) and keloid (*n* = 8) skin samples. The mRNA expression of the extracellular matrix components such as fibronectin and collagen type I were enhanced in keratinocytes from hypertrophic scar lesions (*p* < 0.05 vs. healthy skin; Fig. 1A) and overexpressed in keloid lesions (*p* < 0.05 vs. hypertrophic scar; Fig. 1A). In a similar way, the epithelial cell markers E-cadherin and ZO-1 were decreased in keratinocytes from hypertrophic scar lesions and keloids whereas mesenchymal N-cadherin and αSMA were overexpressed (*p* < 0.05 vs. healthy skin; *p* < 0.05 vs. hypertrophic scar; Fig. 1A) suggesting phenotypic mesenchymal transformation of epidermal keratinocytes in hypertrophic scar and keloid skin lesions. The analysis of PDE4 subtypes showed PDE4B the most overexpressed in keratinocytes from hypertrophic scars and keloids followed by PDE4D expression and in a lesser extent by PDE4A and PDE4C. The immunofluorescence observations supported the overexpression of PDE4B2 in the epidermal keratinocytes together with the increase of αSMA protein expression (Fig. 1B).

### The PDE4 inhibitor roflumilast attenuates the effect of TGFβ on keratinocyte to mesenchymal transition in 3D keratinocytes cell cultures

**Fig. 2:**
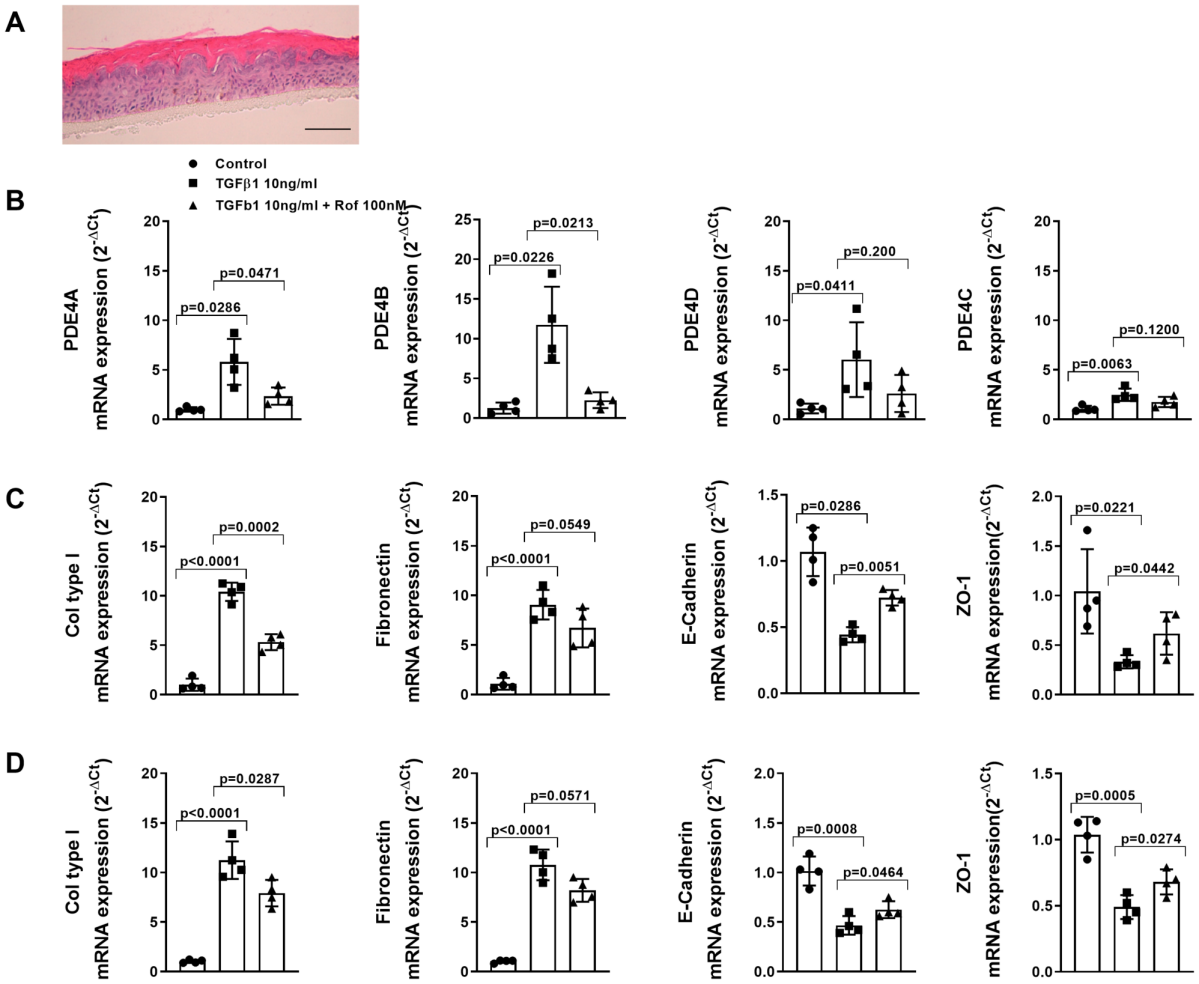
Roflumilast inhibits the increase of phosphodiesterase (PDE) 4 A-D isoforms and epithelial to mesenchymal transition induced by TGFβ1 in 3D epidermis culture. (**A**) Representative image of human 3D epidermis in vitro culture stained with hematoxylin & eosin. (**B**) Human 3D epidermis was incubated with roflumilast (100nM) or vehicle (0.1%DMSO; control) for 30 min followed by the stimulation with TGFβ1 (10 ng/ml) for 48 h. (**C**) Human 3D epidermis was stimulated with TGFβ1 (10 ng/ml) or incubated with vehicle for 48 h, followed by addition of roflumilast (100 nM) or vehicle control (0.1% DMSO) for 30 min and then stimulated again with TGFβ1 (10 ng/ml) for another 48 h. (**D**) Human 3D keratinocytes were initially stimulated for 48 h with TGFβ1 alone followed by the addition of roflumilast 100nM or vehicle and TGFβ1 for another 48 h to evaluate whether roflumilast can reverse the mesenchymal phenotype to keratinocyte (back-differentiation). (**B**, **C** and **D**) Gene mRNA expression of different PDE4 A-D isoforms, collagen type I and fibronectin mesenchymal markers, and epithelial E-cadherin and ZO-1 markers were measured by RT-qPCR and expressed as 2^−ΔCt^. Results are expressed as means ± SE of *n* = 3 independent experiments run in triplicate. Data are presented as scatter dot blot with median and interquartile range values. P-values are based on Kruskal-Wallis test followed by the Dunn’s post-hoc test

Human keratinocytes were differentiated into 3D epidermis on a membrane porous support showing a full basal, columnar and corneus layers (Fig. 2A) as previously we reported (Montero et al. 2022). 3D keratinocytes stimulated with TGFβ1 for 48 h increased the gene expression of the four PDE4 subtypes with a marked overexpression of PDE4B (Fig. 2B). In addition, fibronectin and collagen type I were overexpressed and the epithelial cell markers E-cadherin and ZO-1 were downregulated supporting a mesenchymal transition. In these experimental conditions, roflumilast, added 30 min before TGFβ1 stimulus as a preventive protocol, inhibited the effect of TGFβ1 on PDE4 expression as well as on keratinocyte to mesenchymal markers (Fig. 2C).

To evaluate whether roflumilast can reverse the mesenchymal phenotype to keratinocyte (back-differentiation), 3D keratinocytes were initially stimulated for 48 h with TGFβ1 alone followed by the addition of roflumilast 100nM or vehicle and TGFβ1 for another 48 h (Fig. 2D). Under these conditions, roflumilast reversed mesenchymal markers and rescue keratinocyte markers (Fig. 2D).

**Fig. 3:**
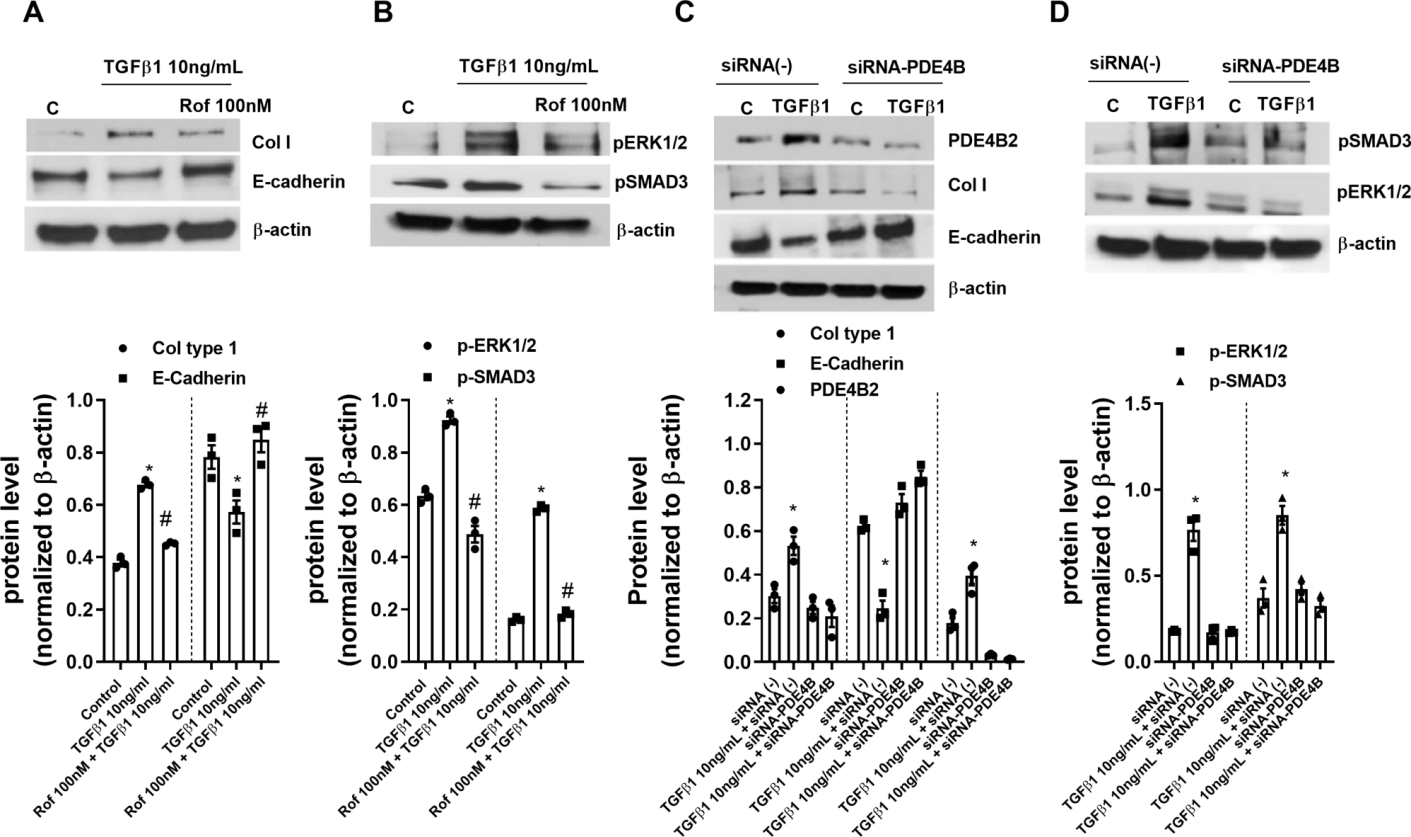
Roflumilast and phosphodiesterase (PDE)4B gene silencing inhibits epithelial to mesenchymal transition, and SMAD2/3 and ERK1/2 phosphorylations induced by TGFβ1 in human keratinocytes. (**A**) Human 3D differentiated epidermis was incubated with roflumilast (Rof, 100nM) or vehicle (0.1% DMSO; control) for 30 min followed by the stimulation with TGFβ1 (10 ng/ml) for 48 h (collagen type I and E cadherin proteins) or 30 min (phosphorylated ERK1/2 and SMAD3 proteins). (**B**) Human keratinocytes cultured in monolayers were transfected with control siRNA(-) or siRNA against PDE4B and then stimulated with TGFβ1 (10ng/ml) for 48 h (PDE4B2, collagen I and E-cadherin) or 30 min (phospho-ERK1/2, phospho-Smad-3). Target proteins and endogenous control (β-actin) were detected by Western blotting. Data are shown as representative Western blots and following densitometric evaluation (ratio between target protein and β-actin) of three independent experiments. Data are presented as scatter dot blot with median and interquartile range values. P-values are based on Kruskal-Wallis test followed by the Dunn’s post-hoc test. *P < 0.05 Control vs. TGFβ1; # *P* < 0.05 symbol TGFβ1 vs. TGFβ1 + Rof

The roflumilast preventive protocol also inhibited the protein expression of collagen type I and rescue E-cadherin to control levels in 3D keratinocytes supporting gene expression findings (Fig. 3A). In parallel, short-time stimulation with TGFβ1 (30 min) increased the phosphorylation of ERK1/2 and SMAD3 which was inhibited by roflumilast in 3D keratinocyte cultures (Fig. 3B).

In other experiments, a potential role of PDE4B in the activation and mesenchymal transition of human keratinocytes was studied (Fig. 3C and D). To this end, keratinocyte monolayer cell cultures were transiently transfected with siRNA against PDE4B or scrambled control siRNA and stimulated with TGFβ1 for 48 h. In the incubations with siRNA control, a ~ 70 kDa PDE4B2 protein was detected at baseline and upregulated following TGFβ1. On the other hand, in the incubations with siRNA against PDE4B the 70 kDa band corresponding to PDE4B2 was decreased both in the absence and presence of TGFβ1 (Fig. 3C). Next, potential effects of the siRNA against PDE4B on TGFβ1-induced keratinocyte-to-mesenchymal transition were explored. Knockdown of PDE4B was accompanied by significantly reduced expression of collagen I and increased expression of E-cadherin following incubation with TGFβ1 over 48 h (Fig. 3C). Given the well-described critical role of TGFβ1-induced Smad-3 and ERK1/2 phosphorylation in epithelial-to-mesenchymal transition, effects of the siRNA against PDE4B on the rapid (30 min) TGFβ1-induced increase in phospho-Smad-3 and phospho-ERK1/2 were investigated next. In fact, knockdown of PDE4B was associated with a significant inhibition of Smad-3 and ERK1/2 phosphorylation (Fig. 3D). Taken together, these findings strongly support a critical role of PDE4B in TGFβ1-induced keratinocyte-to-mesenchymal transition by regulating the phosphorylation status of Smad-3 and ERK1/2 upstream in the TGFβ1 signaling cascade under the current experimental conditions.

### TGFβ1 promotes PDE4B2 and phospho-ERK1/2 complexes that are inhibited by roflumilast in epidermal keratinocytes

**Fig. 4:**
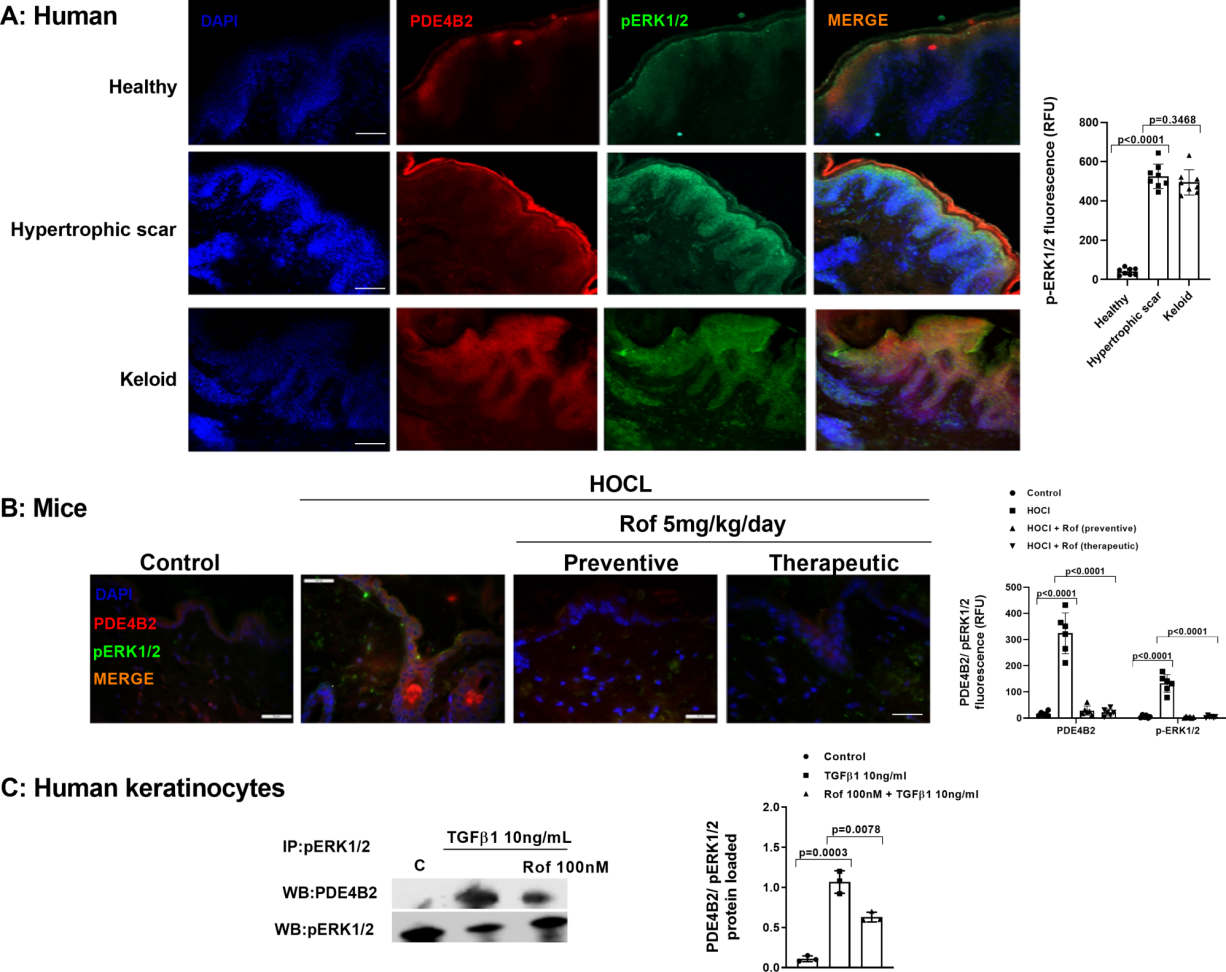
Phosphodiesterase (PDE) 4B2 and phospho-ERK1/2 are co-localized and overexpressed in the epidermis of hypertrophic scar and keloids as well as in hypertrophic epidermis from HOCl-induced skin fibrosis in mice. (**A** and **B**) Immunofluorescence of PDE4B2 (red) and phospho-ERK1/2 (green), their co-expression (orange) and fluorescence intensity measured as relative fluoresce units (RFU) in the epidermal layer. (**A**) Representative images are shown in healthy skin, hypertrophic scar, keloids. Scale bar: 100 μm. (**B**) 24 mice were randomly allocated to three treatment groups (*n* = 10) control (sham), vehicle / HOCl (*n* = 6) and roflumilast (Rof)/ HOCl (*n* = 6). Roflumilast was administered once daily, orally by gavage at 5 mg / kg / d. For preventive treatment, roflumilast was administered from day 1 to day 42. For therapeutic treatment, the PDE4 inhibitor was administered from day 20 to 42. At day 42 mice were sacrificed and Immunofluorescence was performed on altered skin. Scale bar: 50 μm. (**C**) Human keratinocytes were incubated with vehicle (control) or roflumilast (Rof) followed by the stimulation with TGFβ 10ng/ml over 60 min. At the end of stimulation, total protein was extracted and immunoprecipitated (IP) with phospho-ERK1/2 antibody followed by a western blot against PDE4B2. Target proteins and control (phospho-ERK1/2) were detected by Western blotting. Data are shown as representative Western blots and following densitometric evaluation (ratio between target protein and phospho-ERK1/2) of three independent experiments. Data are presented as scatter dot blot with median and interquartile range values. P-values are based on Kruskal-Wallis test followed by the Dunn’s post-hoc test

PDE4B2 and phospho-ERK1/2 were overexpressed in the epidermis layer of keloids and to a lesser extent in hypertrophic scars compared with healthy skin (Fig. 4A), and co-expressed in epidermal keratinocytes. Total ERK1/2 showed similar expression in the epidermis layer of different patients (additional figure S1). Fibrotic skin tissue of animals treated with HOCl showed a protein complex between PDE4B2 and phospho-ERK1/2 in the epidermis as shown in Fig. 4B that was reduced in the animals treated with roflumilast when administered in a preventive or therapeutic protocol (Fig. 4B).

In in vitro experiments, keratinocytes stimulated with TGFβ 10ng/ml over 60 min promoted a protein complex between PDE4B2 and phosphor-ERK1/2 represented by protein immunoprecipitation that was reduced by roflumilast (Fig. 4C).

### TGFβ1 increases NOX4 and reactive oxygen species in dermal fibroblast that are reduced by PDE4 inhibitors

**Fig. 5:**
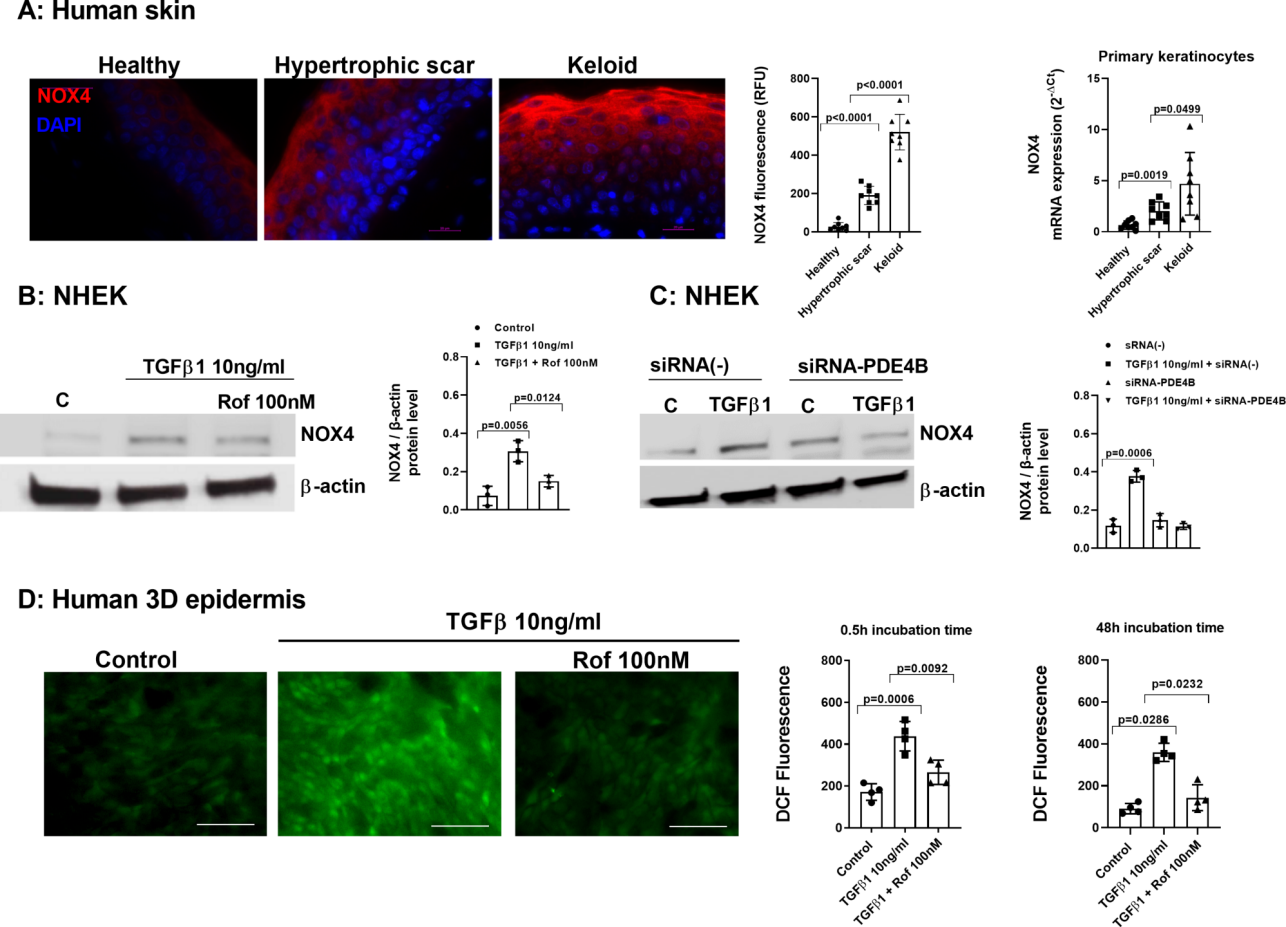
Roflumilast inhibits early and late reactive oxygen species (ROS) release and enhanced NOX4 expression induced by TGFβ1 in normal human dermal fibroblasts (NHDF). (**A**) NOX4 immunofluorescence in epidermis of healthy (*n* = 8), hypertrophic scar (*n* = 8) and keloid (*n* = 8) patients. NOX4 fluorescence intensity measured as relative fluoresce units (RFU) in the epidermal layer. Representative images are shown. Scale bar = 20 μm. NOX4 mRNA expression in primary keratinocytes isolated from epidermis of healthy (*n* = 8), hypertrophic scar (*n* = 8) and keloid (*n* = 8) patients, measured by RT-qPCR and expressed as 2^−ΔCt^. (**B**) Human keratinocytes were incubated for 30 min with roflumilast (Rof, 100 nM) or vehicle, and then stimulated with TGFβ1 (10 ng/ml) for 48 h followed by analysis of NOX4 protein expression by Western blotting. (**C**) Human keratinocytes were transfected with control siRNA(-) or siRNA-PDE4B and stimulated with TGFβ1 (10ng/ml) for 48 h followed by NOX4 protein detection (Western blotting). Single blots, and results from densitometry as the ratio of target protein compared to β-actin. (**D**) Intracellular ROS levels measured by fluorescence microscopy as DCF fluorescence and transformed in arbitrary units after 30 min and 48 h of TGFβ1 (10 ng/ml) stimulation in human 3D differentiated epidermis in the presence or absence of roflumilast (Rof). Scale bar 10 μm. Data are presented as scatter dot blot with median and interquartile range values. P-values are based on Kruskal-Wallis test followed by the Dunn’s post-hoc test

Epidermal keratinocytes from keloids showed an evident overexpression of NOX4 that was also reproduced with lower intensity in epidermal keratinocytes from hypertrophic scars as observed in immunofluorescence and RT-PCR for NOX4 (Fig. 5A).

Normal human epidermal keratinocytes (NHEK) stimulated with TGFβ1 at 10ng/ml for 48 h, showed an increase of NOX4 protein expression (Fig. 5B). Roflumilast (100 nM) or siRNA against PDE4B suppressed the NOX4 increase induced by TGFβ1 (Fig. 5B and C). The elevation of ROS was detected after 30 min and 48 h of TGFβ1 stimulation and inhibited by roflumilast (Fig. 5D).

### Roflumilast inhibits TGFβ1 profibrotic signaling through the inhibition of NOX4/ROS, the increase of PPM1A, and the inhibition of the phosphorylation of SMAD3/ERK1/2

**Fig. 6:**
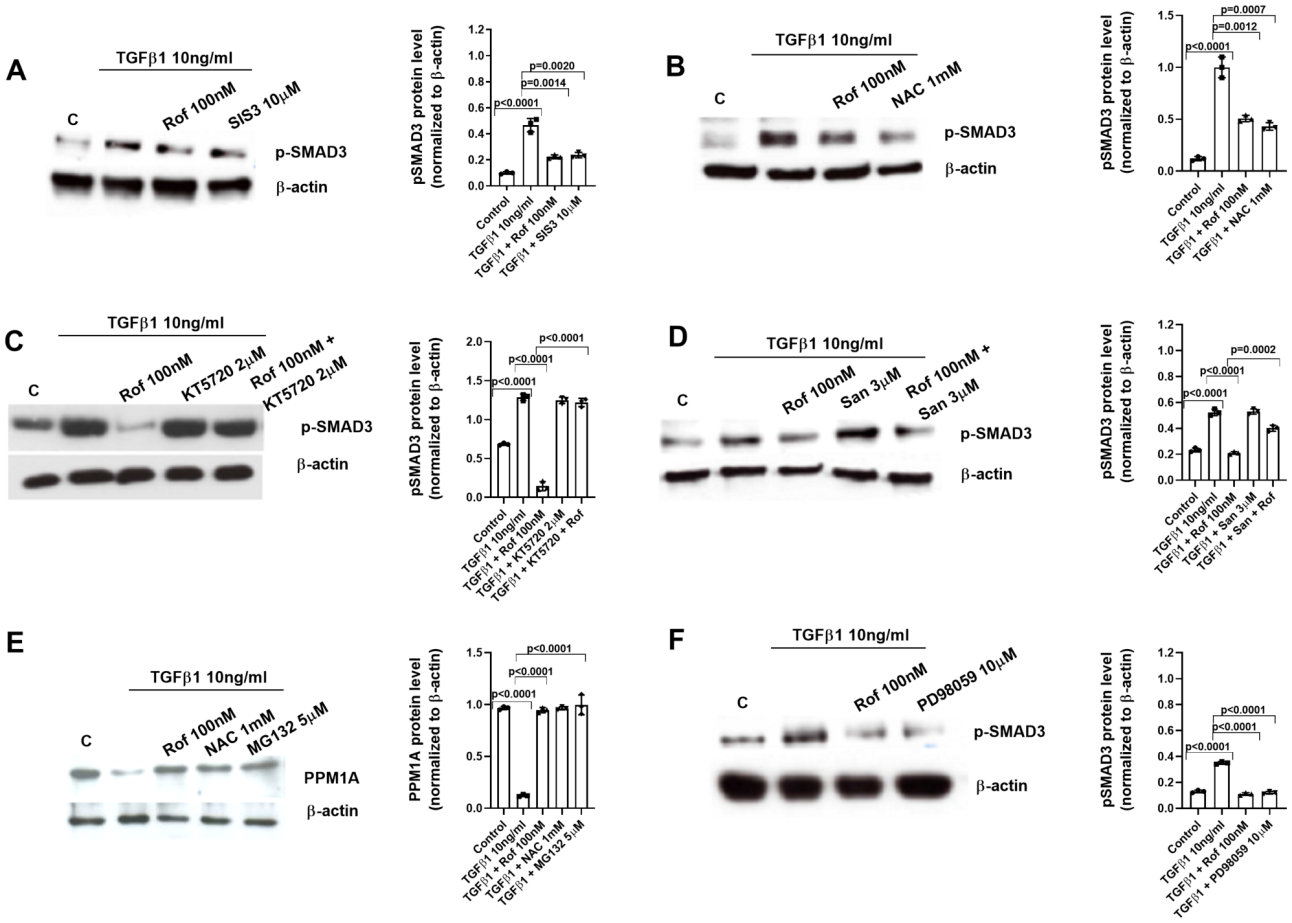
Mechanistic studies (I): Roflumilast prevents TGFβ1-induced phospho-SMAD3 by the increase of PPM1A, the inhibition of reactive oxygen species (ROS) and phospho-ERK1/2-Protein Kinase A axis. Human keratinocytes were incubated for 30 min with vehicle or roflumilast (Rof, 100 nM) (**A**-**F**), the SMAD3 inhibitor (SIS3, 10µM) (**A**), the anti-oxidant N-acetyl-L-cysteine (NAC, 1 mM) (**B**, **E**), the PPM1A inhibitor sanguinarine (San, 3 µM) (**D**), the proteasome inhibitor MG132 (MG132, 5 µM) (**E**), the PKA inhibitor (KT5720, 2µM) (**C**), the ERK1/2 inhibitor (PD98059, 10µM) (**F**) or combinations as indicated. Next, keratinocytes were stimulated with TGFβ1 (10 ng/ml) for 30 min and p-SMAD3 and PPM1A proteins were analysed by Western blotting. Data are shown from densitometry as the ratio of target protein compared to β-actin of three independent experiments per condition. Results of the densitometry are presented as scatter dot blot with median and interquartile range values. P-values are based on Kruskal-Wallis test followed by the Dunn’s post-hoc test

**Fig. 7:**
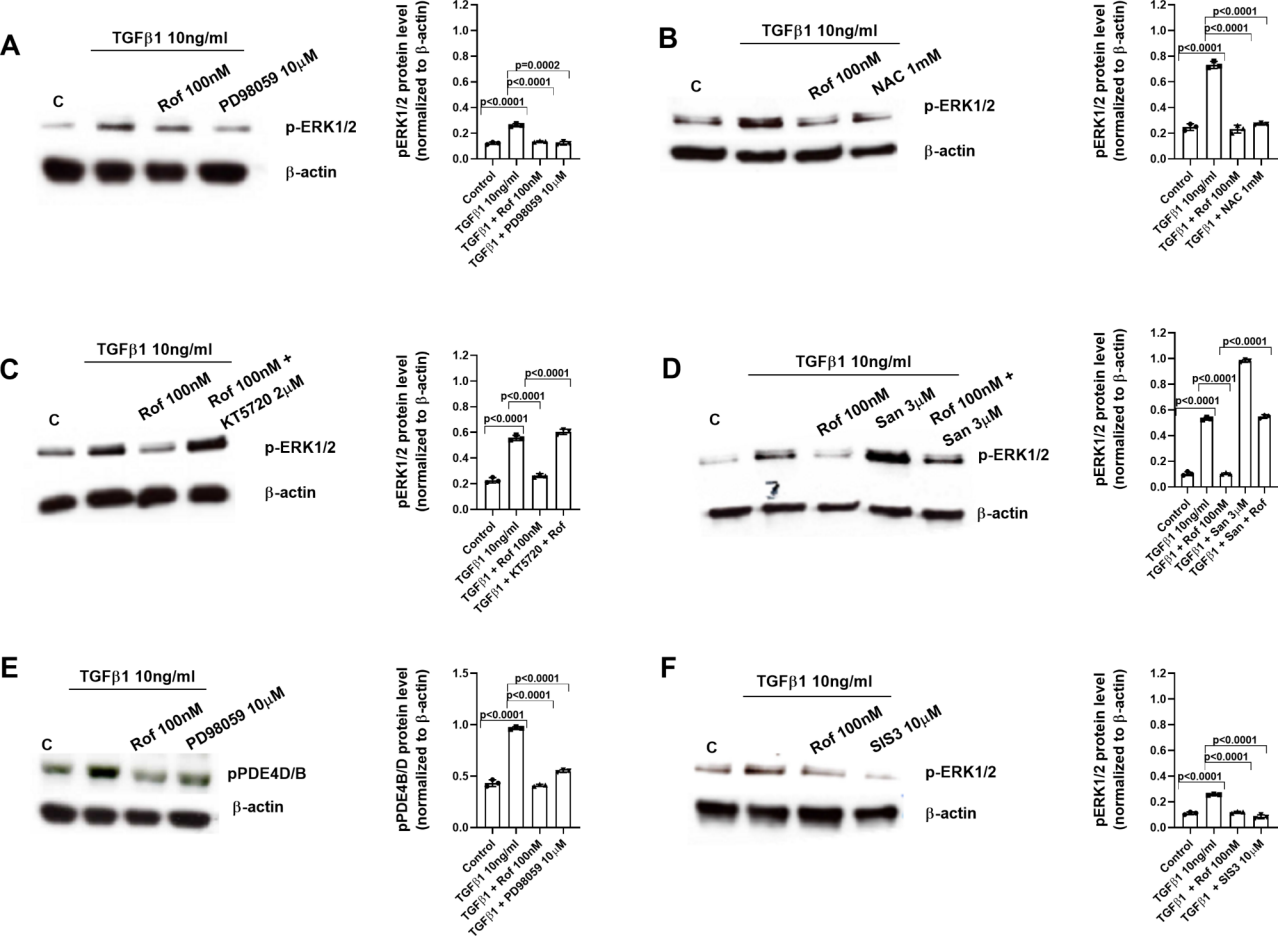
Mechanistic studies (II): Roflumilast prevents TGFβ1-induced phospho-ERK1/2 by the reduction of reactive oxygen species (ROS), the increase of protein kinase A (PKA) and PPM1A. Human keratinocytes were incubated for 30 min with vehicle or roflumilast (Rof, 100 nM) (A-F), the ERK1/2 inhibitor (PD98059, 10µM) (**A**), the anti-oxidant N-acetyl-L-cysteine (NAC, 1 mM) (**B**), the PPM1A inhibitor sanguinarine (San, 3 µM) (**D**), the PKA inhibitor (KT5720, 2µM) (**C**), the SMAD3 inhibitor (SIS3, 10µM) (**F**) or combinations as indicated. Next, keratinocytes were stimulated with TGFβ1 (10 ng/ml) for 30 min and p-SMAD3 and PDE4D/B proteins were analysed by Western blotting. Data are shown from densitometry as the ratio of target protein compared to β-actin of three independent experiments per condition. Results of the densitometry are presented as scatter dot blot with median and interquartile range values. P-values are based on Kruskal-Wallis test followed by the Dunn’s post-hoc test

NHEK were incubated with different inhibitors for 30 min before TGFβ1 (10ng/ml) was added for another 30 min. The levels of phosphorylated SMAD3 (Fig. 6A) and ERK1/2 (Fig. 7A) were measured by western blot. Roflumilast inhibited SMAD3 and ERK1/2 phosphorylations induced by TGFβ1 that were mediated in part by the reduction of ROS since NAC also reduced the SMAD3 and ERK1/2 phosphorylations (Figs. 6B and 7B). As expected, the additional presence of a PKA inhibitor (KT5720, 2 µM) reversed the inhibition of TGFβ1-induced SMAD3 and ERK1/2 phosphorylations by roflumilast (Figs. 6C and 7C). Strikingly, such reversal was also observed with the PPM1A inhibitor sanguinarine (3 µM) (Figs. 6D and 7D). Based on this latter finding the notion may be raised that blocking PDE4 (roflumilast at 100 nM), hence activation of protein kinase A results in an activation of the PPM1A phosphatase activity that has been described to dephosphorylate SMAD3 and ERK1/2 (Figs. 6D and 7D). In parallel experiments, TGFβ1 stimulation during 30 min reduced the levels of PPM1A that were rescued by roflumilast, the anti-oxidant NAC, and the proteasome inhibitor MG132 (Fig. 6E). Furthermore, TGFβ1 increased the phosphorylation of PDE4B/D in the ERK site that was inhibited by roflumilast and the inhibitor of ERK1/2 phosphorylation (Fig. 7E). In addition, we observed that the activation of both SMAD3 or ERK1/2 induced by TGFβ1 can perpetuate the signaling activation by phosphorylation of ERK1/2 and SMAD3 respectively (Figs. 6F and 7F). A representative diagram of this mode of action is presented in Additional figure S2.

### PDE4 inhibition reduces epidermal keratinocyte proliferation and senescence induced by TGFβ1

**Fig. 8:**
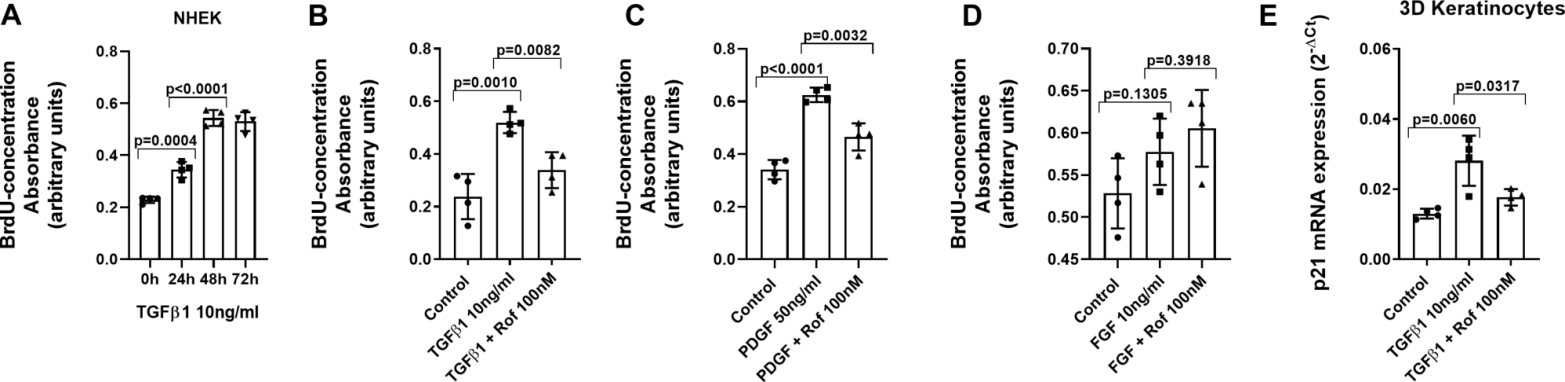
Roflumilast inhibits human keratinocyte proliferation and senescence induced by different growth factors. (**A**-**D**) Human normal keratinocytes were incubated with roflumilast (Rof, 100 nM) or vehicle (0.1% DMSO) for 30 min followed by TGFβ1 (10 ng/ml) (over 24 h, 48 h and 72 h), PDGF (50 ng/ml) or FGF (10 ng/ml) over 24 h. Proliferation was measured using the BrdU incorporation kit. Results are shown as the absorbance at 450 nm from *N* = 4 independent experiments. (**E**) Cellular senescence of human 3D differentiated epidermis was measured after preincubation with roflumilast (Rof, 100 nM) or vehicle control (0.1% DMSO) for 30 min followed by stimulation with TGFβ1 (10 ng/ml) for 72 h, analysing p21 mRNA expression, measured by RT-qPCR and expressed as 2^−ΔCt^. Data are presented as scatter dot blot with median and interquartile range values. P-values are based on Kruskal-Wallis test followed by the Dunn’s post-hoc test

TGFβ1 increased NHEK proliferation in a time-dependent manner, with the highest proliferation observed after 48 h of stimulation, which was maintained at 72 h (Fig. 8A). In subsequent experiments, roflumilast inhibited NHEK proliferation induced by 48 h of TGFβ1 stimulation (Fig. 8B), as well as by other profibrotic growth factors such as PDGF (Fig. 8B), but not FGF (Fig. 8B). After 72 h of TGFβ1 stimulation, the expression of the cell cycle arrest marker P21, an indicator of senescence, was elevated by TGFβ1 and inhibited by roflumilast (Fig. 8E).

### PDE4 inhibition reduced epidermal fibrotic hyperplasia formation induced by subcutaneus HOCl administration in mice

**Fig. 9:**
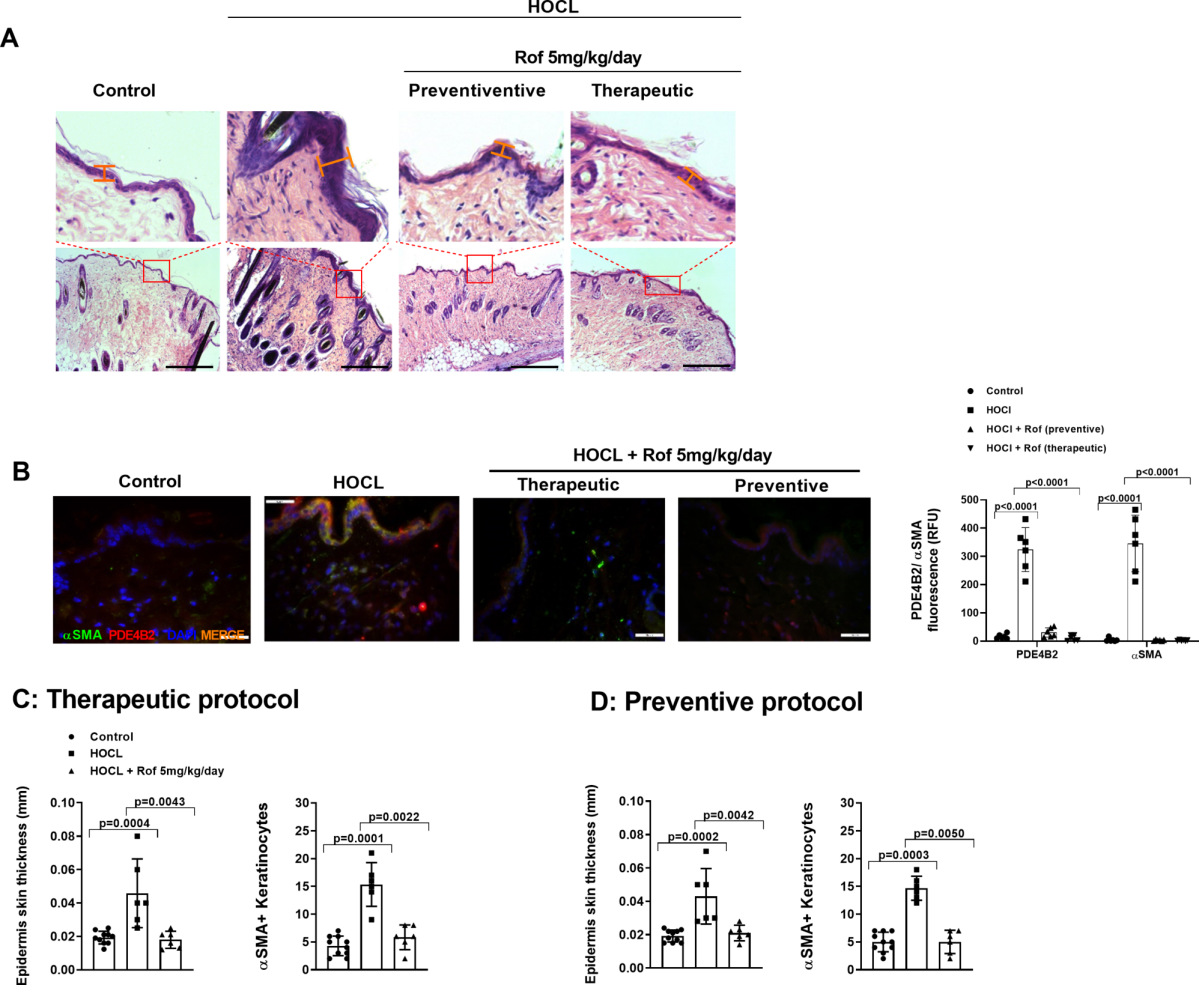
Roflumilast reduced epidermal thickness and αSMA/PDE4B-expressing myofibroblasts in the Cochin chronic oxidant stress model in Balb/c mice. HOCl (10 µg) or vehicle (0.9% physiological saline) in dose volumes of 100 µl were administered subcutaneously, once daily for 42 days to Balb/c mice. Roflumilast (5 mg / kg / d) or vehicle were administered orally between day 0 to 42 day (preventive protocol) or between day 20 to day 42 (therapeutic protocol) of the experimental procedure. A total of *n* = 10, 6, 6 mice in the control, HOCl / Vehicle and HOCl / Roflumilast group, respectively were analysed. (**A**) Hematoxylin & eosin histology of skin fibrotic lesions with respective epidermal region augmentations. (**B**) Immunofluorescence for αSMA (green), PDE4B2 (red), merge (orange) and fluorescence intensity measured as relative fluoresce units (RFU) in the epidermal layer. Representative images are shown. Scale bar: 200 μm in H&E panels and 20 μm in immunofluorescence panels. Skin thickness is indicated with red lines. (**C** and **D**) Epidermis skin thickness and the number of αSMA positive cells per high-power fields (HPF) were measured in the therapeutic protocol (**C**) and preventive protocol (**D**). Data are presented as scatter dot blot with median and interquartile range values. P-values are based on Kruskal-Wallis test followed by the Dunn’s post-hoc test

The HOCl-induced skin fibrosis model in Balb/c mice is a well-described animal model that has been used to characterize anti-fibrotic pharmacology of test compounds (Bagnato et al. 2013; Kotzki et al. 2022; Marut et al. 2013; Morin et al. 2016). Daily subcutaneous administrations of HOCl over 42 days induced local skin fibrosis and epidermal hyperplasia characterized by an increase in skin thickness and αSMA^+^ positive cells (Fig. 9A-D). Roflumilast, at an oral, once daily dose of 5 mg/kg/d in both preventive (administration from day 0–42) and therapeutic (administration from day 20–42) protocols suppressed all of these markers of epithelial hyperplasia (Fig. 8A-D). Representative images from hematoxylin & eosin staining and immunofluorescence studies in fibrotic skin areas showed an increased labeling for epidermal αSMA and PDE4B2 following HOCl versus vehicle that was reduced by roflumilast in both preventive and therapeutic protocols (Fig. 9).

## Discussion

The primary aim of this study is to analyze the involvement of PDE4 in the remodeling of the epidermis observed in skin pathological scars, including hypertrophic scars and keloids. Additionally, we aim to explore the potential of PDE4 inhibition as a promising molecular target for reducing fibrotic skin lesions.

In this study, we present, for the first time, evidence of elevated expression levels of PDE4 isoforms within the hypertrophic epidermis of pathological scars, specifically hypertrophic scars and keloids. Notably, we found that PDE4B is the predominantly expressed isoform distributed among keratinocytes. Furthermore, our results demonstrate that direct inhibition of PDE4 using roflumilast, or silencing of the PDE4B gene, effectively reduces the transition of keratinocytes to myofibroblasts, along with mitigating keratinocyte proliferation and senescence induced by TGFβ1.

In skin keratinocytes, TGFβ1 promotes the phosphorylation of ERK1/2, which subsequently interacts with PDE4B2, leading to its activation through phosphorylation. Inhibition of PDE4 results in increased levels of PKA, effectively reducing ERK1/2 phosphorylation. Moreover, both roflumilast and siRNA targeting PDE4B decrease the levels of NOX4 and ROS in keratinocytes, consequently elevating the levels of PPM1A. This increase in PPM1A inhibits SMAD3 and ERK1/2 phosphorylation, thereby counteracting the pro-fibrotic effects induced by TGFβ1 (Additional Figure S2). The anti-fibrotic effects of PDE4 inhibition on the skin were also evaluated in a mouse model of HOCl-induced skin fibrosis, thereby confirming the potential of targeting PDE4 to mitigate the expansion of pathological scars.

Hypertrophic scars and keloids are primarily attributed to the uncontrolled formation of excessive extracellular matrix (ECM) and fibroblast proliferation during the wound healing process. Although the exact mechanism underlying pathological scar formation remains incompletely understood, recent studies have identified several contributing factors. These include skin trauma, genetic predisposition, chronic inflammation subsequent to initial skin injury, and the release of pro-fibrotic growth factors such as platelet-derived growth factor (PDGF), vascular endothelial growth factor (VEGF), and TGFβ. These factors contribute to the activation, proliferation, and differentiation of skin myofibroblasts, ultimately leading to the excessive deposition of ECM. Among these factors, the TGFβ1/SMAD3 canonical pathway is the most extensively characterized and potent inducer of fibrosis in pathological scars (Andrews et al. 2016). Current treatments for pathological scars are limited and restricted to silicone dressing, surgical removal and intralesional triamcinolone acetonide injections between others (Yang et al. 2021), with different degrees of efficacy and recurrences (Cheraghi et al. 2017). Therefore, there is an unmet need to understand the mechanisms involved in pathological scars formation to develop new potential treatments.

Several recent studies suggest that the fibrosis in hypertrophic scars involves the epithelial mesenchymal transition (EMT) (Yuan et al. 2019; Ong et al. 2007; Yan et al. 2010, 2015). During the initial phases of skin wound healing, myofibroblasts originating from EMT facilitate wound contraction and extracellular matrix (ECM) secretion (Yuan et al. 2019; Radisky et al. 2007). Nevertheless, during later stages, unresolved inflammation can impede EMT progression, potentially resulting in scar formation. Hence, EMT emerges as a crucial factor in orchestrating skin wound healing processes.

EMT is characterized by the loss of keratinocyte features, including the reduction of epithelial markers E-cadherin and ZO-1, and the adoption of mesenchymal characteristics, such as increased expression of αSMA, ECM proteins, and N-cadherin, among others. In our investigation, we noted a pronounced upregulation of mesenchymal markers within the epidermal layer, including fibronectin, collagen type I, αSMA (a cytoskeletal protein), and mesenchymal N-cadherin. This dysregulation exhibited a gradient with keloid epidermis showing the most significant alteration, followed by hypertrophic scar epidermis and then healthy epidermis. These findings corroborate earlier studies (Yuan et al. 2019; Yan et al. 2015), further substantiating the involvement of EMT in keloids and hypertrophic scars.

Earlier findings suggest that the cAMP second messenger system plays a crucial role in modulating fibrotic processes. Inhibiting PDE4, an enzyme responsible for degrading cAMP, shows promise in mitigating lung fibrosis by targeting epithelial alveolar type II and lung fibroblast cells (Cortijo et al. 2009; Milara et al. 2014a, b, 2015; Hatzelmann et al. 2010). In skin disorders, the PDE4 inhibitor apremilast, reduces the activation of M1/M2 macrophages, T-cells, and fibroblast skin accumulation in various animal models of systemic sclerosis (SSc) (Lu et al. 2021; Maier et al. 2017) without direct effect on skin fibroblasts from SSc patients (Maier et al. 2017). However, the presence and function of PDE4 in pathological scars remain unknown to our knowledge. In psoriatic disease, apremilast has been shown to inhibit dermal fibroblast activation and myofibroblast transition (Schafer et al. 2016). Interestingly, all PDE4 isoforms are expressed in skin lesions of psoriatic arthritis, atopic dermatitis, and discoid lupus erythematosus, with PDE4B and PDE4D being the most prominent in normal dermal fibroblasts (Schafer et al. 2016). Furthermore, PDE4 isoforms are overexpressed in epidermal skin biopsies of patients with atopic dermatitis, and apremilast significantly reduces inflammation and remodeling of atopic epidermis in vitro and in animal models of atopic dermatitis, suggesting an anti-inflammatory role of PDE4 inhibitors in inflammatory skin diseases by targeting keratinocytes (Schafer et al. 2019). In our study, we observed that PDE4B was the most abundantly expressed isoform in the epidermis of hypertrophic scars and keloids, followed by PDE4D. This discovery is noteworthy, given that novel drugs targeting PDE4B inhibition have demonstrated anti-fibrotic properties (Sgalla et al. 2021), while also exhibiting improved safety profiles compared to commonly used non-selective PDE4 inhibitors like roflumilast and apremilast, which are often associated with gastrointestinal side effects (Contreras et al. 2017).

This observation led us to investigate the role of PDE4 in epidermal activation and transformation to mesenchymal-like myofibroblasts. Using in vitro differentiated 3D human epidermis, complete and selective inhibition of PDE4 (100 nM roflumilast) almost abrogated an increase of PDE4 isoforms, as well as mesenchymal markers collagen I and fibronectin. The decreased expression of keratinocyte markers E-cadherin and ZO-1 induced by TGFβ1 were also inhibited by roflumilast in both myofibroblast differentiation and dedifferentiation protocols in line with a previous report in skin fibroblasts where PDE4 was blocked with rolipram or apremilast (Cutolo et al. 2020). As we observed with roflumilast, the selective siRNA-PDE4B reduced epidermal EMT markers, as well as the signaling pathways induced by TGFβ1 such as p-ERK1/2 and p-SMAD3.

In addition, roflumilast inhibited keratinocyte proliferation and senescence, thus indicating a prominent role of PDE4 on epidermal remodeling associated with skin fibrosis. Similar results have been detected in lung fibroblast, were siRNA-PDE4B inhibited TGFβ1-induced FMT and bFGF-induced fibroblast proliferation (Selige et al. 2011).

Dermal fibroblast senescence has been observed in keloids, but no data is available on keratinocytes. Previous findings showed that P16 senescence marker was overexpressed in αSMA positive dermal fibroblasts in hypertrophic scar and keloid tissue suggesting a key role on pathological scar (Limandjaja et al. 2020). Senescent fibroblast have been detected in different fibrotic organs, characterized by the inability to further proliferate, acquiring an inflammatory phenotype (labelled senescence-associated secretory phenotype or SASP), largely characterized by overexpression of pro-inflammatory cytokines and growth factors such as FGF, PDGF or TGFβ, which stimulate non-senescent cells (Arvia et al. 2021). In this work, we observed that chronic TGFβ1 stimulation induce 3D human epidermal senescence characterized by the p21 overexpression, that was alleviated by roflumilast. Senescence promoted by TGFβ1 is mediated in part by the canonical SMAD2/3 signaling and oxidative stress generation mediated by NOX4 (Hubackova et al. 2012). NOX4 (NADPH oxidase 4) is part of the NADPH oxidase enzyme family, which generates ROS by transferring electrons from NADPH to oxygen. NOX4 has been implicated in the development of skin fibrosis (Qin et al. 2022). It contributes to the fibrotic process primarily through the production of ROS. Elevated ROS levels can induce fibroblast activation and differentiation into myofibroblasts, which are central to the fibrotic response. ROS also promote the production of pro-fibrotic cytokines and growth factors, further amplifying the fibrotic process. Increased NOX4 expression has been observed in the skin of patients with systemic sclerosis and in experimental models of skin fibrosis (Piera-Velazquez et al. 2015). The enzyme’s activity is linked to key fibrotic pathways, including those involving TGF-β, a major regulator of fibrosis (Piera-Velazquez and Jimenez 2021). By generating ROS, NOX4 influences TGF-β signaling and other pathways that drive fibrosis. Given its role in skin fibrosis, NOX4 is considered a potential therapeutic target and several NOX4 antibodies are under research (Thannickal et al. 2023). Indeed, keloid tissue exhibits heightened oxidative stress concurrent with the downregulation of the Nrf2 antioxidant transcription factor (Lee et al. 2015). However, the mechanisms that produce oxidative stress in pathological scar is limited. Here, we observed for the first time that keloid epidermis and in a lesser extent hypertrophic scar epidermis express large amounts of NOX4, and that TGFβ1 induces the expression of NOX4 and reactive oxygen species which is inhibited by roflumilast and siRNA-PDE4B. These results may explain, almost in part, the effects of PDE4 inhibition on keratinocyte senescence.

To further explain the anti-fibrotic effects of PDE4 inhibition in human keratinocytes, we observed that roflumilast elevated the levels of PPM1A in epidermal keratinocytes, probably by the inhibition of oxidative stress, since PPM1A is blocked by oxidative stress (Jiang et al. 2014). As previously described, PPM1A is a phosphatase that can dephosphorylate SMAD2/3 and ERK1/2 inhibiting canonical and non-canonical TGFβ pathways (Jiang et al. 2014; Li et al. 2014) and therefore the fibrotic processes such as EMT.

Prior studies have demonstrated that apremilast inhibits dermal fibroblast-to-myofibroblast transition (FMT) by suppressing SMAD3 and ERK1/2 phosphorylation (Cutolo et al. 2020), yet the precise mechanism underlying this inhibition remains unclear. Here, we propose that the downregulation of NOX4 and reduction of oxidative stress, along with the upregulation of PPM1A, contribute to the dephosphorylation of SMAD3 and ERK1/2 induced by PDE4 inhibition. In addition, we observed that TGFβ1 increases PDE4B2 in 3D epidermis. Between different PDE4B isoforms, it appears that short PDE4B2 is the inducible isoform, and induced by oxidative stress in inflammatory cells (Barber et al. 2004) while large PDE4B1,3 are more constitutive, which support our findings. In this work, the addition of roflumilast elevated PKA in epidermal keratinocytes.

Prior studies have demonstrated that PKA can inhibit ERK1/2 phosphorylation (Pabbidi et al. 2016). Furthermore, the phosphorylation of ERK1/2 can trigger the phosphorylation of PDE4B2 at the ERK site within its catalytic domain, enhancing its function and reducing cAMP levels (Baillie et al. 2000; MacKenzie et al. 2000). Consequently, this creates a positive feedback loop, diminishing PKA activity while elevating ERK1/2 phosphorylation.

In this work we observed that roflumilast reduced phospho-ERK1/2-PDE4B2 complexes and diminished the effect of TGFβ on PDE4B/D phosphorylation at ERK catalytic domain, breaking the positive phospho-ERK1/2 feedback mechanism.

To corroborate in vitro findings, a mouse model of s.c HOCl-induced skin fibrosis was used. In this model, HOCl provoked an epidermal hypertrophy with an increase of epidermal αSMA^+^ cells in the site of HOCl administration. Furthermore, PDE4B2 expression was increased in epidermal keratinocytes and co-localized with phopho-ERK1/2 and αSMA positive cells confirming in vitro findings. The oral administration of roflumilast in a preventive and therapeutic protocols was able to reduce epidermal thickness and epidermal αSMA^+^ cells. However, although in vivo results can provide information about the effects of PDE4 inhibition on pathological scar, the HOCl model is characterized by systemic inflammation producing fibrosis in other tissues such as kidney or lung, more relevant to SSc (Bagnato et al. 2013), which may be interpreted as a limitation of this study.

## Conclusions

In summary, this is the first report that characterize the role of PDE4 in the remodeled epidermis of pathological scars such as hypertrophic scar and keloids, showing that PDE4B inhibition could be an attractive target to attenuate the growth of the skin fibrotic lesion.

### Electronic supplementary material

Below is the link to the electronic supplementary material.

Supplementary Material 1

## Data Availability

All data generated or analysed during this study are included in this published article [and its supplementary information files].

## Abreviations

ECM: extracellular matrix
EMT: Epithelial to mesenchymal transition
FMT: fibroblast-to-myofibroblast transition
PDE4: Phosphodiesterase 4
PDGF: platelet-derived growth factor
ROS: reactive oxygen species
siRNA: small interfering RNA
SSc: systemic sclerosis
TGFβ: Transforming growth factor beta
VEGF: vascular endothelial growth factor
