# Supplementary material for: Phosphodiesterase 4 is overexpressed in keloid epidermal scars and its inhibition reduces keratinocyte fibrotic alterations
Source: Mol Med. 2024 Sep 2;30:134. doi: 10.1186/s10020-024-00906-8 (PMC11370283; doi:10.1186/s10020-024-00906-8)
Supplement: Supplementary file 1 — Supplementary Material 1 [file 10020_2024_906_MOESM1_ESM.docx]

**SUPPLEMENTARY MATERIAL**

**Title**

**Phosphodiesterase 4 is overexpressed in keloid epidermal scars and its inhibition reduces keratinocyte fibrotic alterations.**

**Authors**

**Javier Milara^1,2,3*^, Pilar Ribera^2^*, Severiano Marín^4^, Paula Montero^2,5^, Inés Roger^1,2,5^, Julio Cortijo^1,2^**

**Author Affiliations**

^1^ CIBER de enfermedades respiratorias, Health Institute Carlos III, Valencia, Spain.

^2^ Department of Pharmacology, Faculty of Medicine, University of Valencia, Spain.

^3^ Pharmacy unit, University General Hospital Consortium of Valencia, Spain.

^4^Plastic Surgery Unit, University General Hospital Consortium, 46014 Valencia, Spain

^5^Faculty of health sciences, Universidad Europea de Valencia, 46010, Valencia, Spain

*Both authors contributed equally to this work

**Corresponding author:**

Javier Milara, PhD, PharmD; Department of Pharmacology, Faculty of Medicine, University of Valencia, Avenida Blasco Ibáñez, 15. 46010 Valencia. Spain. Tel. +43-96 386 41 00; eMail: xmilara@hotmail.com

**ADDITIONAL FIGURES**

**Additional figure S1. Total expression of ERK1/2 in the epidermis of healthy control, hypertrophic scar and keloids.** Immunofluorescence of ERK1/2 (green). Representative images are shown in healthy skin, hypertrophic scar and keloids. Scale bar: 100 µm.


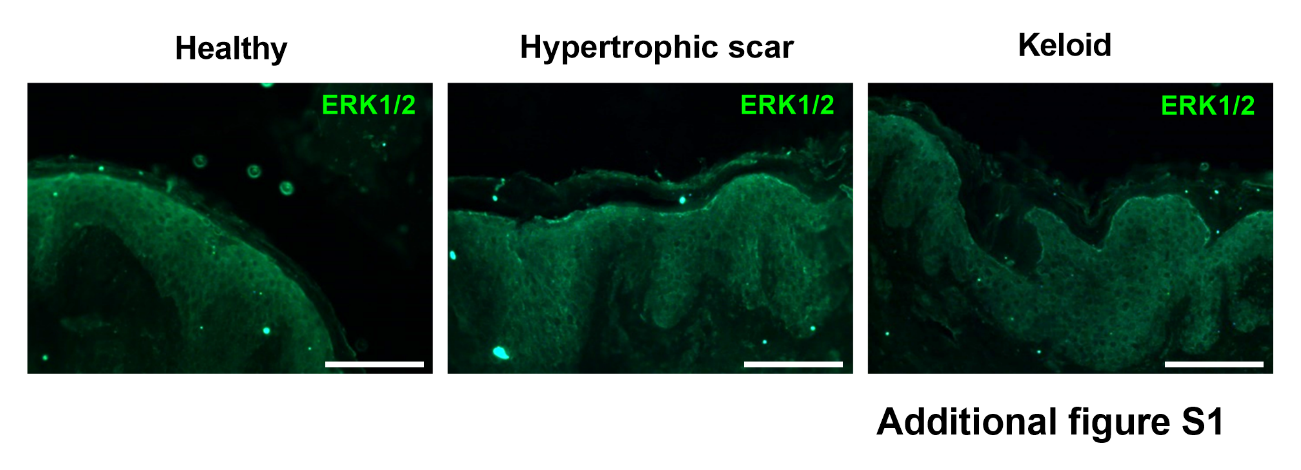


**Additional figure S2. Schematic representation of phosphodiesterase (PDE)4/B inhibition on keratinocyte to mesenchymal transition process**. TGFβ1 activates canonical SMAD2/3 and noncanonical ERK1/2 pathways. Both, phosphorylations of SMAD2/3 and ERK1/2 promote the gene expression of mesenchymal-like myofibroblast markers, senescence and oxidative stress/NOX4 armament. Phosphorylated ERK1/2 activates PDE4B reducing the levels of cAMP and protein kinase A (PKA). On the other hand, TGFβ1 increases reactive oxygen species (ROS) and later expression of NOX4 which reduce the levels of protein phosphatase, Mg^2+^/Mn^2+^ Dependent 1A (PPM1A). Low levels of PKA (an inhibitor of ERK1/2) and PPM1A (phosphatase that dephosphorylates SMAD2/3) allow SMAD2/3 and ERK1/2 overactivation, thus increasing the TGFβ1-induced cell activation and the process of epithelial to mesenchymal transition and fibrosis. Roflumilast, a PDE4A-D inhibitor, and selective PDE4B gene silencing reduces ROS/NOX4 expression, increases PPM1A and PKA, reducing the levels of SMAD2/3 and ERK1/2 phosphorylation, inhibiting TGFβ1-induced epidermal remodeling.


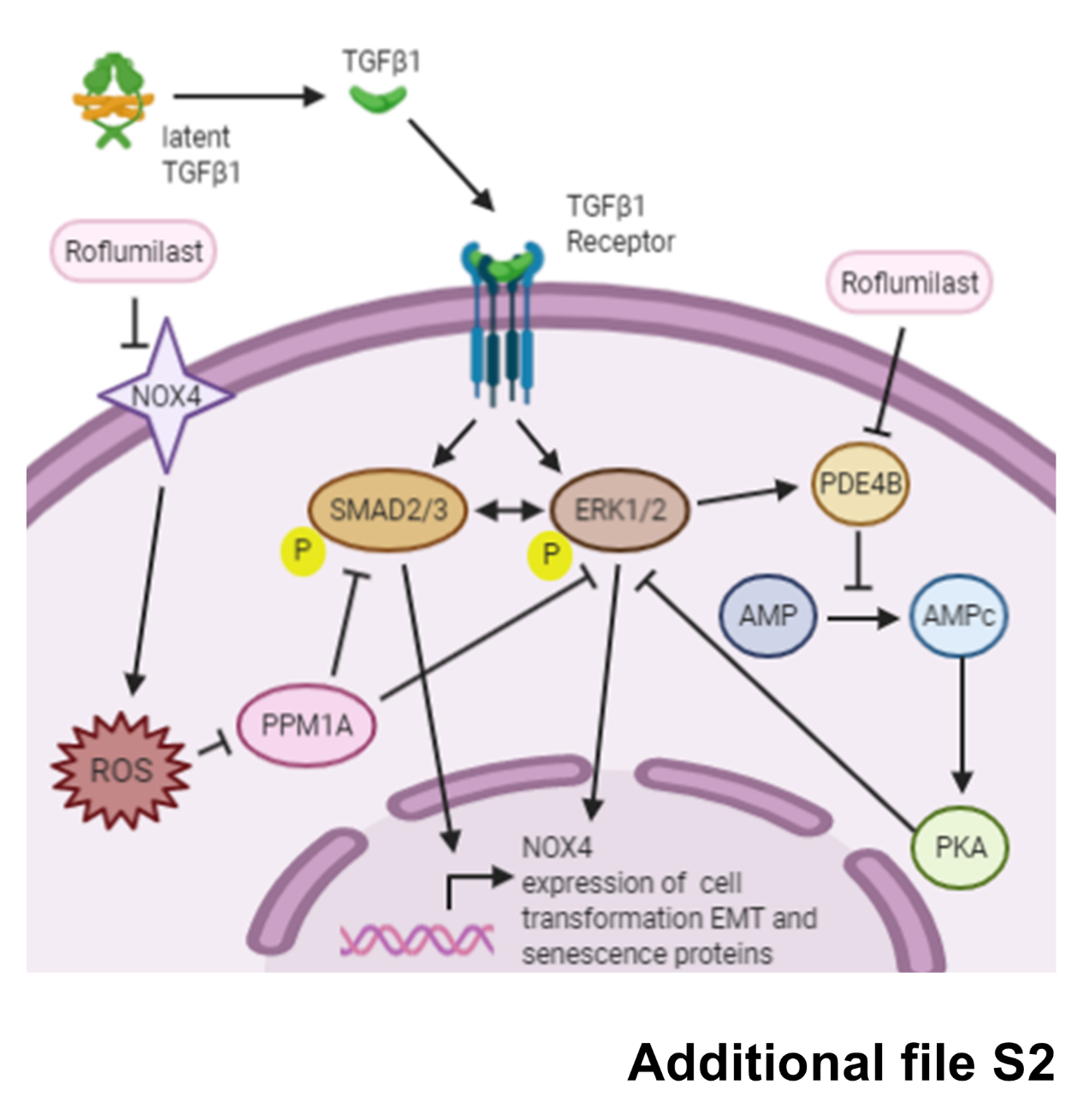


**Additional Table S1.**

Table S1. Demographic data of patients from whom redundant tissue specimens from normal skin, hypertrophic scars and keloids were obtained. K: Keloid; HS: Hypertrophic scar; H: Healthy.

| **Patient ID** | **Tissue** | **Body site** | **Sex** | **Age** | **Ethnicity** |
| --- | --- | --- | --- | --- | --- |
| 1K | Keloid | Chest | M | 35 | Caucasian |
| 2K | Keloid | Neck | M | 53 | Caucasian |
| 3K | Keloid | Chest | M | 49 | Caucasian |
| 4K | Keloid | Chest | M | 56 | Caucasian |
| 5K | Keloid | Abdomen | M | 47 | Caucasian |
| 6K | Keloid | Neck | M | 53 | Caucasian |
| 7K | Keloid | Neck | F | 55 | Caucasian |
| 8K | Keloid | Neck | F | 48 | Caucasian |
| 1HS | Hypertrophic scar | Upper limb | M | 31 | Caucasian |
| 2HS | Hypertrophic scar | Chest | M | 40 | Caucasian |
| 3HS | Hypertrophic scar | Chest | M | 51 | Caucasian |
| 4HS | Hypertrophic scar | Abdomen | M | 38 | Caucasian |
| 5HS | Hypertrophic scar | Abdomen | F | 46 | Caucasian |
| 6HS | Hypertrophic scar | Neck | F | 49 | Caucasian |
| 7HS | Hypertrophic scar | Abdomen | M | 43 | Caucasian |
| 8HS | Hypertrophic scar | Abdomen | F | 48 | Caucasian |
| 1H | Normal human skin | Abdomen | M | 34 | Caucasian |
| 2H | Normal human skin | Abdomen | M | 56 | Caucasian |
| 3H | Normal human skin | Abdomen | M | 45 | Caucasian |
| 4H | Normal human skin | Abdomen | M | 48 | Caucasian |
| 5H | Normal human skin | Abdomen | M | 52 | Caucasian |
| 6H | Normal human skin | Abdomen | M | 43 | Caucasian |
| 7H | Normal human skin | Abdomen | F | 37 | Caucasian |
| 8H | Normal human skin | Abdomen | F | 49 | Caucasian |
